# Involvement of the miR‐128‐3p/KDM3A/NLRP3 Axis in High Glucose‐Induced Inflammatory Injury in Retinal Endothelial Cells

**DOI:** 10.1002/kjm2.70120

**Published:** 2025-09-25

**Authors:** Wei‐Ming Wen, Jian‐Bo Feng, Yin‐Sheng Cai, Nan Lin, Fei Lv, Zhu‐Sheng Guo

**Affiliations:** ^1^ Department of Clinical Laboratory Dongguan Tungwah Hospital Dongguan China; ^2^ Department of Clinical Laboratory Dongguan SongShan Lake Tungwah Hospital Dongguan China

**Keywords:** diabetic retinopathy, H3K9me2, inflammatory injury, KDM3A, miR‐128‐3p

## Abstract

This study explores the regulatory mechanism of the miR‐128‐3p in diabetic retinopathy (DR)‐associated inflammatory injury. A cellular model of DR was established by inducing immortalized human retinal endothelial cells (IM‐HRECs) with high‐glucose (HG). Cell viability was evaluated by CCK‐8 assay, and the levels of TNF‐α, IL‐1β, and IL‐10 were measured by ELISA. RT‐qPCR was performed to determine miR‐128‐3p expression, and miR‐128‐3p mimics were transfected into cells to verify its regulatory role in DR‐associated inflammatory injury. miR‐128‐3p was predicted by Starbase to bind to the 3′ UTR of KDM3A, which was verified by dual‐luciferase assay. The expressions of KDM3A and NLRP3 in cells were examined by Western blotting, and the enrichment of KDM3A and H3K9me2 on the NLRP3 promoter was measured by Ch‐IP assay. The results revealed that HG treatment significantly reduced both IM‐HREC viability and IL‐10 levels, increased the levels of TNF‐α and IL‐1β, and downregulated the expression of miR‐128‐3p. Overexpression of miR‐128‐3p reduced inflammation in IM‐HRECs induced by HG. The proposed mechanism involves targeting of the KDM3A 3′ UTR by miR‐128‐3p, leading to reduced KDM3A expression, while KDM3A increased NLRP3 expression by reducing H3K9me2. In conclusion, upregulation of miR‐128‐3p increases the histone H3K9me2 level by inhibiting KDM3A expression, thereby reducing NLRP3 expression and suppressing DR inflammatory injury.

## 1 Introduction

Diabetic retinopathy (DR) is a major ocular complication of diabetes mellitus, causing damage to the neurons and microvasculature of the retina and ultimately leading to vision deficits [1]. The condition has long been considered a disease of the microvasculature due to observed abnormalities in the retinal microvasculature, although recent evidence suggests that the multiple molecular and physiological changes seen in the DR‐affected retina are consistent with the presence of inflammation [2, 3]. An accumulation of hyperglycemic damage disrupts the delicate balance in the retina, leading to increased production of pro‐inflammatory mediators [4]. Chronic low‐grade inflammation has been found in both animal models of diabetes and patients at different stages of DR [5]. In the streptozotocin‐induced diabetic mouse model, inhibition of the microglial‐initiated inflammatory response alleviates DR [6]. Therefore, the elucidation of the underlying inflammatory pathogenesis of DR may have promising therapeutic results.

microRNAs (miRNAs) are small endogenous non‐coding RNAs (approximately 21 nucleotides in length) that serve as post‐transcriptional regulators of gene expression. Dysregulation of miRNAs has been implicated in various pathophysiological processes associated with DR [7, 8], including glucose homeostasis, angiogenesis, and the inflammatory response [9]. One miRNA, miR‐128‐3p, has been found to be weakly expressed in HG‐treated retinal vascular endothelial cells and retinal tissues of DR mice [10], while overexpression of miR‐128‐3p enhances the viability of HG‐treated retinal pigment epithelial cells and lowers the inflammatory response [11]. Moreover, overexpression of miR‐128‐3p has also been shown to reduce inflammation associated with LPS‐induced sepsis [12, 13], while miR‐128‐3p knockdown aggravates hemin‐induced inflammation in BV2 cells and neurotoxicity linked to intracerebral hemorrhage [14]. These findings prompted us to investigate the specific mechanism underlying the effects of miR‐128‐3p in DR‐associated inflammation.

Lysine specific demethylase 3 A (KDM3A) is an epigenetic activator influencing the transcription of target genes by removing the suppressive histone dimethylation mark of histone H3 at lysine 9 (H3K9me2) [15]. KDM3A expression is upregulated in the vascular and cardiac tissues in type 2 diabetes, while downregulation of KDM3A significantly alleviates diabetic myocardial injury in diabetic rats and also reduces vascular smooth muscle cell dysfunction induced by high glucose in vitro [16]. Inhibition of KDM3A can epigenetically modify NF‐κB/p65 by increasing the H3K9me2 level, mitigating hyperglycemia‐mediated myocardial injury and cardiac dysfunction [17]. The benefits of KDM3A in reducing high insulin‐induced dysfunction of vascular smooth muscle cells have also been demonstrated, resulting from its ability to modulate inflammation, apoptosis, and oxidative stress [18]. However, the precise role of KDM3A in DR‐associated inflammatory injury has not been identified.

Nucleotide‐binding domain, leucine‐rich‐containing family, pyrin domain‐containing‐3 (NLRP3) is a member of the NLR family of innate immune cell sensors, which responds to exogenous pathogenic invasion and endogenous cellular damage by initiation of the inflammatory response [19]. Activation of the NLRP3 inflammasome underlies the pathogenesis of diabetes and its complications via modulation of glucose tolerance, insulin resistance, inflammation, and pyroptosis [20, 21]. Patients with proliferative DR show elevated NLRP3 expression in their fibrovascular membranes [22]. As DR progresses, excessive activation of NLRP3 triggers further inflammation and retinal cell death, resulting in an inflammatory storm that incurs structural and functional collapse of neurovascular units, ultimately impairing vision [23]. The present study hypothesizes that miR‐128‐3p regulates high glucose (HG)‐induced inflammatory injury in retinal endothelial cells via the KDM3A/NLRP3 axis. This study reveals the involvement of the miR‐128‐3p/KDM3A/NLRP3 axis in HG‐induced inflammatory injury in retinal endothelial cells to provide a theoretical basis for the treatment of DR.

## 2 Materials and Methods

### 2.1 Cell Culture and Treatment

Immortalized human retinal endothelial cells (IM‐HRECs) were procured from Innoprot (P10880‐IM, Innoprot, Derio, Bbzkaia, Spain) and were cultured in Dulbecco's Modified Eagle Medium (DMEM) (Invitrogen, Waltham, MA, USA) containing 10% fetal bovine serum (FBS, PAA Laboratories GmbH, Pasching, Austria) and 1% penicillin–streptomycin (Invitrogen) at 37°C with 5% CO_2_.

Glucose (HY‐B0389, MedChemExpress, Monmouth Junction, NJ, USA) was dissolved in ultrapure water to form a stock solution of 1 M. Before use, the HG solution was diluted to 30 mM with DMEM and used to treat cells for 24 h to construct an HG model. Cells treated with 5 mM glucose for 24 h were used as a control group.

KDM3A and NLRP3 were inserted into the pcDNA3.1 vector (Thermo Fisher Scientific, Waltham, MA, USA) to obtain the overexpression plasmids, KDM3A (oe‐KDM3A) and NLRP3 (oe‐NLRP3). IM‐HREC cells were grown until 80% confluent and were then transfected with the miR‐128‐3p mimic (miR‐mimic), mimic NC, oe‐NC, oe‐KDM3A, and oe‐NLRP3 plasmids using Lipofectamine 3000 reagent (Thermo Fisher Scientific). The miR‐mimic, oe‐KDM3A, oe‐NLRP3, and their corresponding controls were provided by GenePharma (Shanghai, China). After 48 h of transfection, the cells were harvested for further experiments.

### 2.2 RT‐qPCR

Total RNA was extracted from IM‐HREC cells using TRIzol reagent (Takara, Shiga, Japan) and was reverse‐transcribed into cDNA using a PrimeScript RT reagent kit with gDNA Eraser (RR047A, Takara). miRNA was synthesized into cDNA using an MiR‐X miRNA First‐Strand Synthesis Kit (638,315, Takara). RT‐qPCR was performed using TB Green Premix Ex Taq II (RR820A, Takara) on an ABI 7500 real‐time PCR system thermocycler (Applied Biosystems, Waltham, MA, USA). β‐actin and U6 [24] were used as the internal references, and relative gene expression was calculated by the 2^−ΔΔCt^ method [25]. The primer sequences are shown in Table 1.

**TABLE 1:** PCR primer sequence.

Name	Sequence (5′‐3′)
miR‐128‐3p	F: CGCGTCACAGTGAACCGGT
	R: AGTGCAGGGTCCGAGGTATT
KDM3A	F: CTGCCCTTGTTCAAACAGGC
	R: GTGAGGAGTGTCAAAGCCCA
NLRP3	F: CGGGGCCTCTTTTCAGTTCT
	R: TTGTCTCCGAGAGTGTTGCC
β‐Actin	F: AAGGACTCCTATAGTGGGTGACGA
	R: ATCTTCTCCATGTCGTCCCAGTTG
U6	F: CGAGCACAGAATCGCTTCA
	R: CTCGCTTCGGCAGCACATAT
NLRP3 promoter	F: ATTTGGGCTCTACGTGTGCA
	R: TGGAAAACGGTGGAGCTCTC

*Note*: miR‐128‐3p: microRNA‐128‐3p; KDM3A: Lysine‐specific demethylase 3A; NLRP3: Nucleotide‐binding oligomerization domain‐like receptor protein 3.

### 2.3 Cell Counting Kit‐8 (CCK‐8) Assay

Cell viability was assessed using a CCK‐8 assay kit (CK04, Dojindo Molecular Technologies, Tokyo, Japan). Briefly, cells were seeded into 96‐well plates and cultured at 37°C for 24 h. After transfection, the cells were cultured for a further 48 h and were then treated with 10 μL of CCK‐8 solution per well for 1 h. The absorbance at 450 nm was measured.

### 2.4 Enzyme‐Linked Immunosorbent Assay (ELISA)

The culture supernatants from the different groups of cells were collected. The levels of TNF‐α, IL‐1β, and IL‐10 were measured using ELISA kits (ab285312/ab217608/ab185986, Abcam, Cambridge, UK), as directed. The absorbance at 450 nm was read, and the expression was calculated according to the standard curve.

### 2.5 Dual Luciferase Assay

KDM3A 3′UTR fragments containing wild‐type or mutant binding sites of miR‐128‐3p were inserted into the pmirGLO plasmid (E1330, Promega, Madison, WI, USA) to construct the WT and MUT plasmids, which were co‐transfected with mimic NC or miR‐128‐3p mimic (miR‐mimic) into cells. After 48 h, the cells were collected and lysed. A luciferase assay kit (K801‐200, BioVision, Mountain View, CA, USA) was used to detect the luciferase activity.

### 2.6 Western Blotting

Cells were lysed with RIPA buffer containing protease inhibitors (Boster Biological Technology Co. Ltd. Wuhan, Hubei, China), and protein concentrations were measured by BCA assays (AR1189, Boster Biological Technology Co. Ltd). The proteins were then separated on 10% SDS‐PAGE gels, followed by transfer to polyvinylidene fluoride (PVDF) membranes (T2234, Thermo Fisher Scientific) and blocking with 5% BSA for 2 h. Then, the membrane was incubated overnight at 4°C with primary rabbit monoclonal antibodies against KDM3A (ab191389, 1:1000, Abcam) and NLRP3 (ab263899, 1:1000, Abcam), as well as a rabbit polyclonal antibody against β‐actin (ab8227, 1:1000, Abcam). After washing, the membrane was treated with goat anti‐rabbit secondary antibody IgG (ab205718, 1:2000, Abcam) at room temperature for 1 h. Enhanced chemical fluorescence (34,580, Thermo Fisher Scientific) was used to visualize the bands, and grayscale quantification was performed using ImageJ software (NIH, Bethesda, MA, USA).

### 2.7 Chromatin Immunoprecipitation (Ch‐IP)

Ch‐IP was performed using a Ch‐IP Kit (# 56383, Cell Signaling Technology, Danvers, MA, USA). Briefly, the cells were fixed with formaldehyde solution for 10 min and then lysed. The nuclear protein complex was treated with ultrasound and then incubated with antibodies against IgG (ab171870, Abcam), KDM3A (sc‐376,608X, Santa Cruz, Dallas, TX, USA), and H3K9me2 (ab176882, Abcam). The following day, the complexes were mixed with protein A/G magnetic beads and precipitated at 4°C for 4 h. The immunoprecipitated DNA was purified using the QIAquick PCR Purification Kit (28,106, Qiagen, Germany) and analyzed by RT‐qPCR.

### 2.8 Statistical Analysis

Data analysis and graph plotting were performed using SPSS 21.0 (IBM Corp. Armonk, NY, USA) and GraphPad Prism 8.0 (GraphPad Software, San Diego, CA, USA). The data were examined for normal distribution and homogeneity of variance. The measurement data are expressed as mean ± standard deviation and were analyzed using *t*‐tests for comparisons between two groups, and one‐way or two‐way analysis of variance (ANOVA) for comparisons among multiple groups, following Tukey's multiple comparison test. A value of *p* < 0.05 indicated a significant difference.

## 3 Results

### 3.1 Overexpression of miR‐128‐3p Alleviates HG‐induced DR inflammatory injury

**FIGURE 1:**
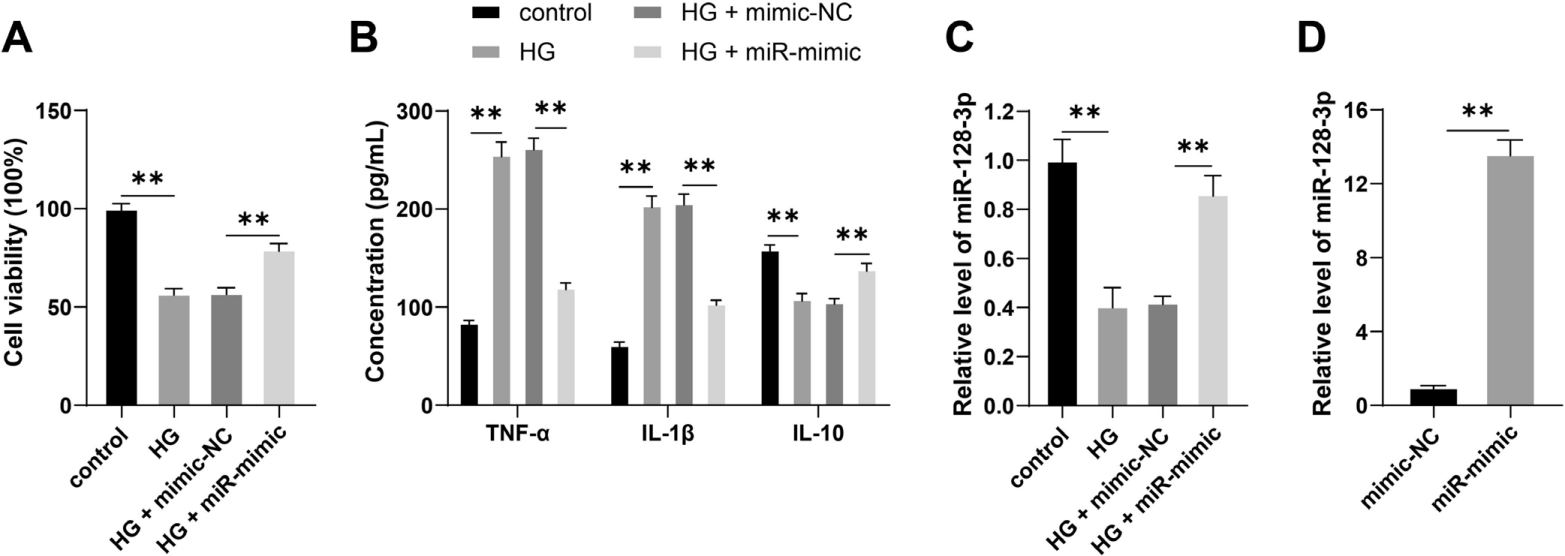
Overexpression of miR‐128‐3p alleviates HG‐induced DR inflammatory injury. Immortalized‐human retinal endothelial cells (IM‐HRECs) were treated with 5 mM or 30 mM glucose for 24 h. (A): Cell viability, assessed by CCK‐8 assays; B: TNF‐α, IL‐1β, and IL‐10 levels in cells, measured by ELISA; (C): Expression of miR‐128‐3p in cells, measured by RT‐qPCR. Then cells were transfected with an miR‐128‐3p mimic (miR‐mimic), with mimic‐NC as a control. D: Transfection efficiency, measured by RT‐qPCR. Experiments were repeated three times independently. The data are expressed as mean ± standard deviation. The data in panels (A) and (C) were analyzed by one‐way ANOVA, and the data in panel (B) were analyzed by two‐way ANOVA. The data in panel (D) were analyzed by independent‐samples *t*‐tests, followed by Tukey's multiple comparisons test. **p < 0.01.

An in vitro model of DR was established by treating IM‐HREC cells with HG. Compared with the control group, HG treatment significantly reduced both cell viability and IL‐10 expression in IM‐HREC cells (*p* < 0.01, Figure 1A,B), and significantly elevated the levels of TNF‐α and IL‐1 β (*p* < 0.01, Figure 1B), indicating that HG treatment induced DR inflammatory injury. Meanwhile, the RT‐qPCR results revealed that miR‐128‐3p expression was significantly lower in the HG group than that in the control group (*p* < 0.01, Figure 1C), suggesting that miR‐128‐3p may be a potential therapeutic target for DR.

To further verify the regulatory role of miR‐128‐3p in DR inflammatory injury, we upregulated miR‐128‐3p expression in IM‐HRECs (*p* < 0.01, Figure 1C,D). Overexpression of miR‐128‐3p significantly increased both viability and IL‐10 expression in HG‐treated cells (*p* < 0.01, Figure 1A,B), while the levels of TNF‐α and IL‐1β were significantly reduced (*p* < 0.01, Figure 1B), indicating that overexpression of miR‐128‐3p alleviates HG‐induced DR inflammatory injury.

### 3.2 miR‐128‐3p Modulates KDM3A Expression

**FIGURE 2:**
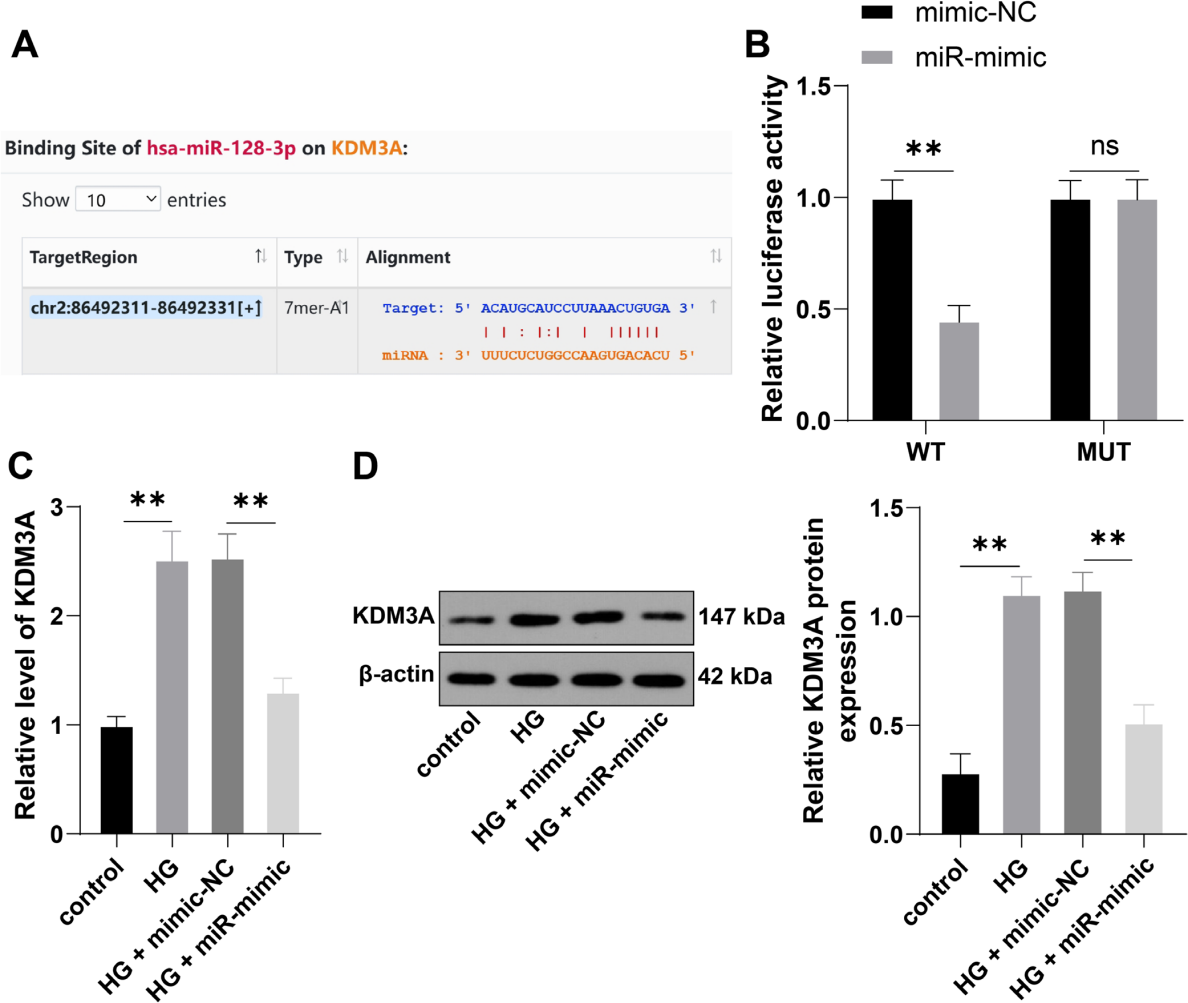
miR‐128‐3p targets KDM3A expression. (A): The binding site of miR‐128‐3p to the KDM3A 3′UTR, predicted by Starbase; (B): Verification of binding between miR‐128‐3p and KDM3A, shown by dual‐luciferase reporter assay; (C): KDM3A expression in cells, as shown by RT‐qPCR (C) and Western blotting (D). The cell experiments were repeated three times independently. The data are expressed as mean ± standard deviation. The data in panel (B) were analyzed by two‐way ANOVA, and the data in panels (C, D) were analyzed by one‐way ANOVA, followed by Tukey's multiple comparisons test. ns *p* > 0.05, **p < 0.01.

To examine the downstream mechanism of miR‐128‐3p, we predicted through the Starbase that miR‐128‐3p had a potential binding to the KDM3A 3′ UTR, and a dual‐luciferase assay verified the binding between miR‐128‐3p and KDM3A (*p* < 0.01, Figure 2A,B). RT‐qPCR and Western blotting results showed that KDM3A expression in the HG‐treated group was markedly increased (*p* < 0.01), while its expression was significantly reduced by the overexpression of miR‐128‐3p (*p* < 0.01, Figure 2C,D). These results indicate that miR‐128‐3p can bind to KDM3A and inhibit its expression.

### 3.3 Overexpression of KDM3A Partially Reverses the Inhibitory Effect of miR‐128‐3p Overexpression on DR Inflammatory Injury

**FIGURE 3:**
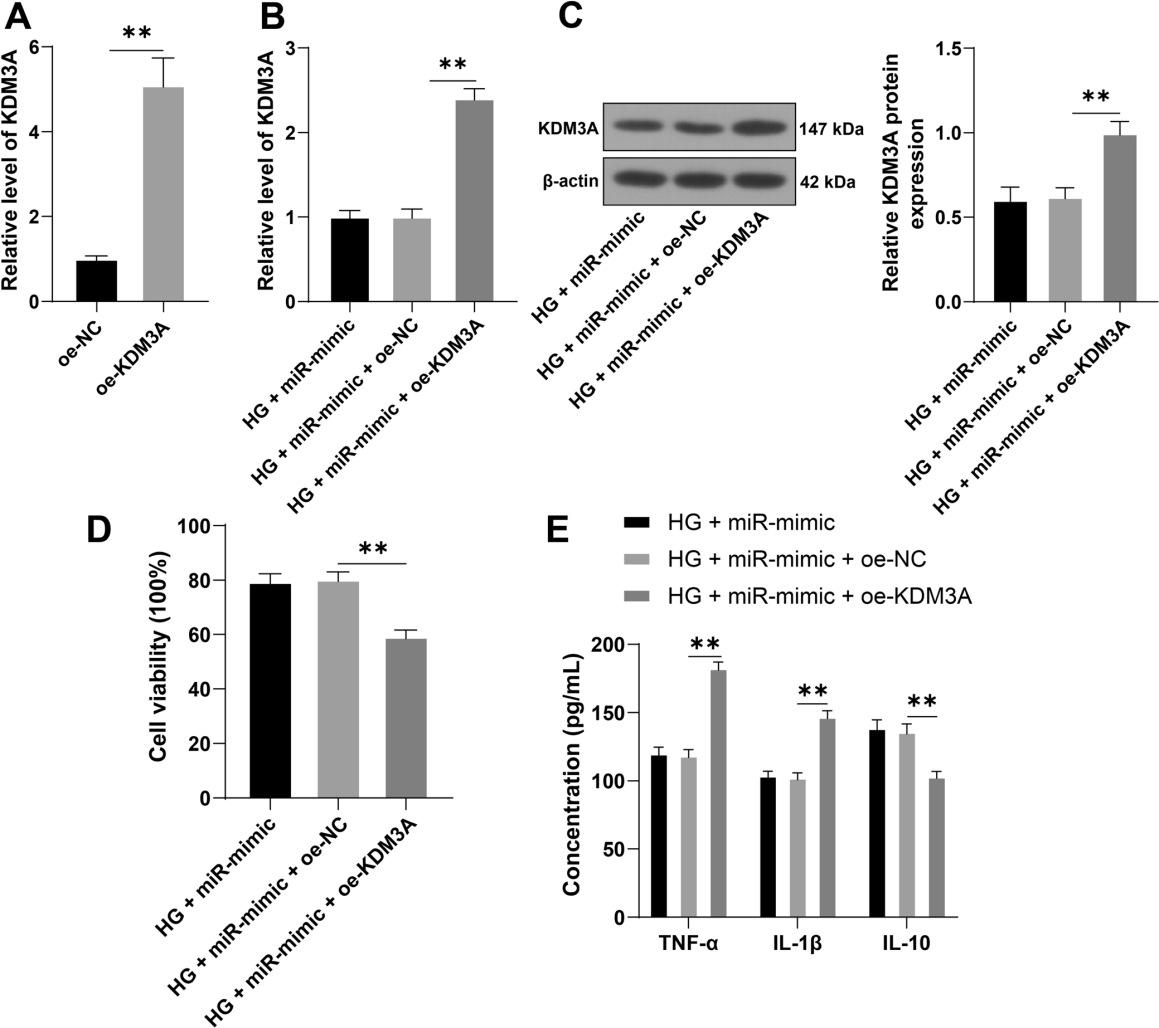
Overexpression of KDM3A partially reverses the inhibitory effect of miR‐128‐3p overexpression on DR inflammatory injury. The KDM3A overexpression plasmid (oe‐KDM3A) was transfected into IM‐HRECs, using oe‐NC as a control, followed by a combined treatment with the miR‐128‐3p mimic (miR‐mimic) under high‐glucose conditions. (A, B): KDM3A mRNA expression, as shown by RT‐qPCR; (C): KDM3A protein expression in cells, as shown by Western blotting; (D): Cell viability, as shown by CCK‐8 assays; (E): TNF‐α, IL‐1β, and IL‐10 levels in cells, as measured by ELISA. The cell experiments were repeated three times independently. The data are expressed as mean ± standard deviation. The data in panel (A) were analyzed by independent‐samples t test. The data in panels (B–D) were analyzed by one‐way ANOVA, and the data in panel (E) were analyzed by two‐way ANOVA, followed by Tukey's multiple comparisons test. **p < 0.01.

To verify the above regulatory mechanism, we upregulated KDM3A expression in cells (*p* < 0.01, Figure 3A), followed by a combined experiment with the miR‐128‐3p mimic (miR‐mimic). Compared with transfection with miR‐mimic alone under HG treatment, KDM3A expression was significantly increased in the combined treatment group (*p* < 0.01, Figure 3B,C), while cell viability and IL‐10 expression were markedly reduced (*p* < 0.01, Figure 3D,E), and the levels of TNF‐α and IL‐1β were significantly elevated (*p* < 0.01, Figure 3E). These results indicate that overexpression of KDM3A could partially reverse the inhibitory effect of miR‐128‐3p overexpression on DR inflammatory injury.

### 3.4 KDM3A Promotes Transcription and Expression of NLRP3 by Removal of Histone H3K9me2

**FIGURE 4:**
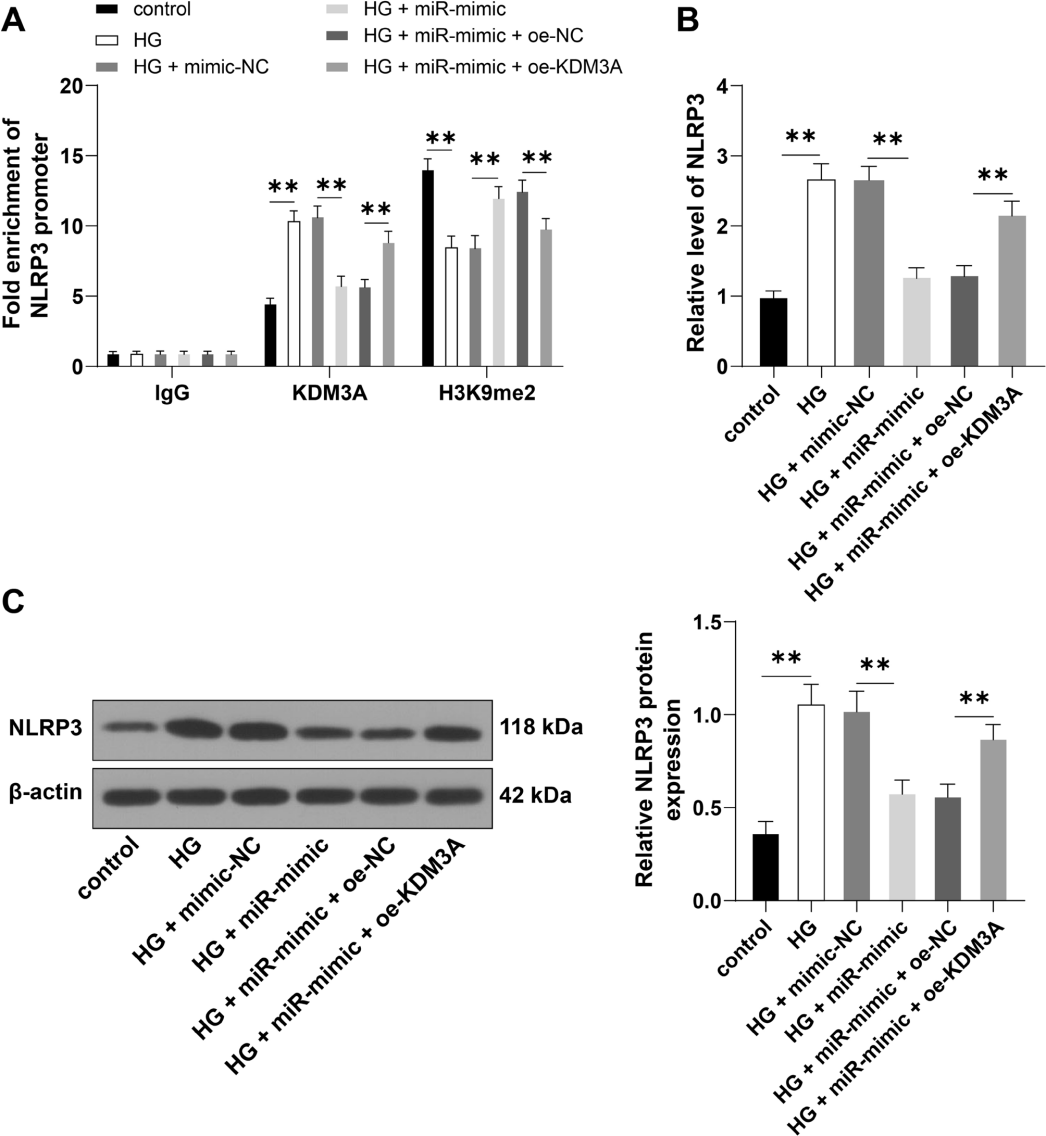
KDM3A promotes NLRP3 expression by removal of histone H3K9me2. (A): Enrichment of KDM3A and H3K9me2 on the NLRP3 promoter region in cells, as shown by Ch‐IP; (B, C): NLRP3 expression in cells, as measured by RT‐qPCR (B) and Western blotting (C). The cell experiments were repeated three times independently. The data are expressed as mean ± standard deviation. The data in panel (A) were analyzed by two‐way ANOVA, and the data in panels (B–C) were analyzed by one‐way ANOVA, followed by Tukey's multiple comparisons test. **p < 0.01.

KDM3A is a histone demethylase that promotes the transcription of downstream target genes by the removal of H3K9me2 [26]. NLRP3 is an important component of the NLRP3 inflammasome [27]. It was hypothesized that KDM3A was involved in the DR inflammatory process by regulating NLRP3 expression through histone H3K9me2 modification. Ch‐IP results showed that KDM3A enrichment was enhanced on the NLRP3 promoter after HG treatment (*p* < 0.01) but reduced after the overexpression of miR‐128‐3p (*p* < 0.01), while the enrichment of H3K9me2 on the NLRP3 promoter showed the opposite trend (*p* < 0.01). In addition, overexpression of KDM3A increased the enrichment of KDM3A on the NLRP3 promoter, while reducing the enrichment of H3K9me2 (*p* < 0.01, Figure 4A). RT‐qPCR and Western blotting results demonstrated that both the mRNA and protein levels of NLRP3 in the HG treatment group were significantly higher than those in the control group (*p* < 0.01). Overexpression of miR‐128‐3p reduced both mRNA and protein levels of NLRP3 (*p* < 0.01), while oe‐KDM3A increased elevated the transcription and expression levels of NLRP3 expression at both the mRNA and protein levels (*p* < 0.01, Figure 4B,C). The above results indicate that KDM3A promotes the transcription and protein expression of NLRP3 by the removal of histone H3K9me2.

### 3.5 Overexpression of NLRP3 Partially Reverses the Inhibitory Effect of miR‐128‐3p Overexpression on DR Inflammatory Injury

**FIGURE 5:**
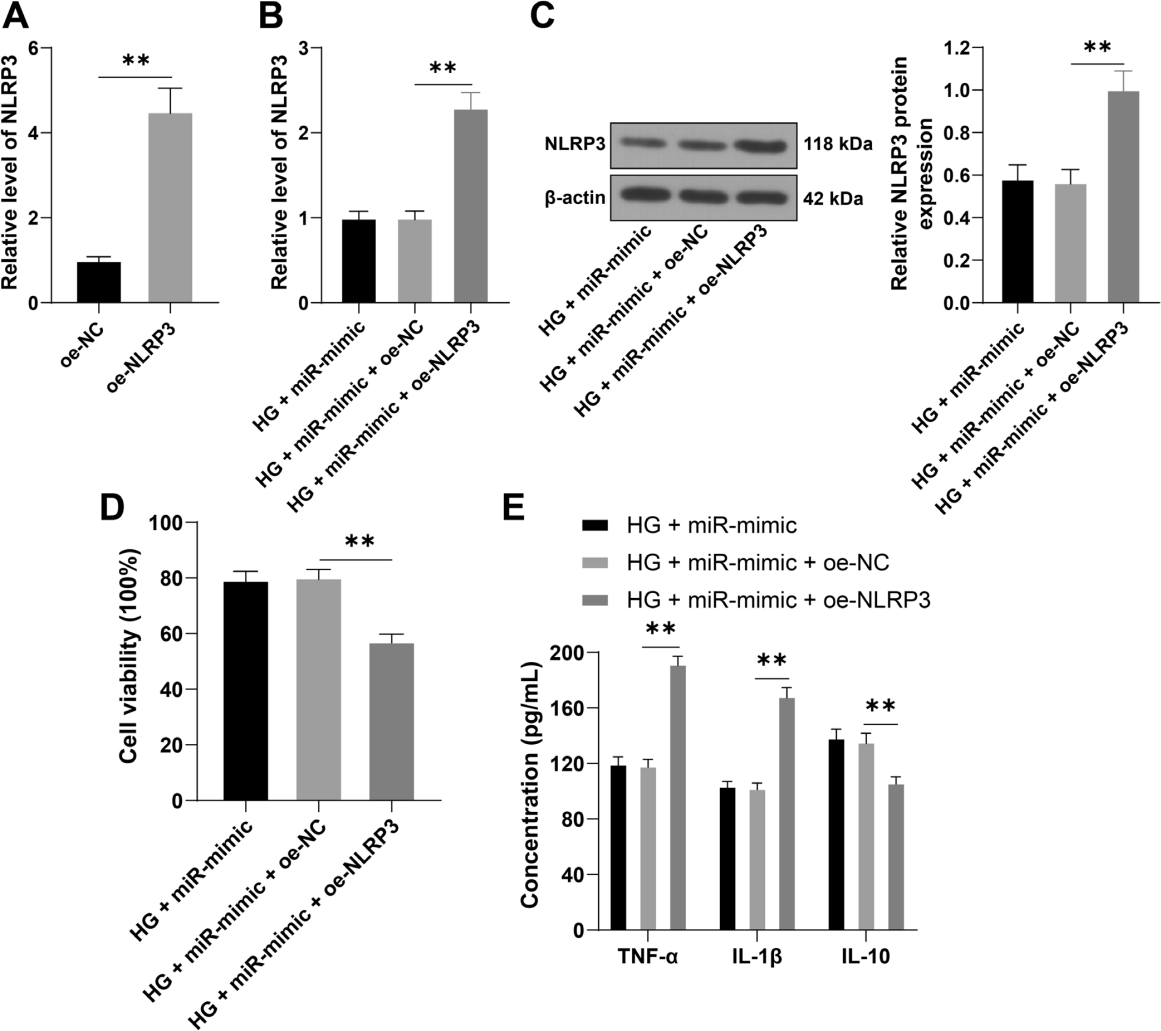
Overexpression of NLRP3 partially reverses the inhibitory effect of miR‐128‐3p overexpression on DR inflammatory injury. The NLRP3 overexpression plasmid (oe‐NLRP3) was transfected into IM‐HRECs, with oe‐NC as a control, followed by combined treatment with the miR‐128‐3p mimic (miR‐mimic) under high‐glucose conditions. (A, B): NLRP3 mRNA expression in cells, as shown by RT‐qPCR; (C): NLRP3 protein expression in cells, as shown by Western blotting; (D): Cell viability, as shown by CCK‐8 assays; (E): TNF‐α, IL‐1β, and IL‐10 levels in cells, as shown by ELISA. The cell experiments were repeated 3 times independently. The data are expressed as mean ± standard deviation. The data in panel (A) were analyzed by independent‐samples t test. The data in panels (B–D) were analyzed by one‐way ANOVA, and the data in panel (E) were analyzed by two‐way ANOVA, followed by Tukey's multiple comparisons test. **p < 0.01.

To further verify the above mechanism, we upregulated NLRP3 expression in cells (*p* < 0.01, Figure 5A–C), followed by a combined experiment with the miR‐128‐3p mimic (miR‐mimic) under HG treatment. Compared with the transfection of miR‐mimic alone, cell viability and IL‐10 expression were significantly decreased in the combined treatment group (*p* < 0.01, Figure 5D,E), along with significantly increased levels of TNF‐α and IL‐1β (*p* < 0.01, Figure 5E). The above results indicate that miR‐128‐3p alleviates DR inflammatory injury by suppressing the expression of NLRP3.

## 4 Discussion

DR represents a major cause of visual impairment among working‐age people worldwide. The pathogenesis of DR is complex, and inflammation has been widely shown to mediate DR‐associated structural and molecular changes [3]. Convincing evidence emphasizes the vital role of miRNAs in regulating key processes associated with DR development, including angiogenesis and inflammation [8]. This study demonstrated that miR‐128‐3p alleviated HG‐induced inflammatory injury in retinal endothelial cells via the KDM3A/NLRP3 axis.

Chronic inflammation in diabetes may be destructive, resulting in irreversible injury to fragile tissues such as the retina [5]. Inflammatory cytokines, including TNF‐α, IL‐6, IL‐8, and IL‐1β, are markedly raised in diabetic patients, and a high degree of inflammation indicates a more severe DR condition [5, 28]. We simulated DR in vitro by treating IM‐HREC cells with HG and found that HG induction reduced both cell viability and IL‐10 expression while significantly elevating TNF‐α and IL‐1β, indicating that HG treatment aggravated DR inflammatory injury. miR‐128‐3p plays protective or deleterious roles in different diseases. Herein, we found decreased miR‐128‐3p expression in HG‐induced cells, overexpression of miR‐128‐3p rescued HG‐induced reduction of cell viability and IL‐10 expression, and upregulation of IL‐1β and TNF‐α in IM‐HRECs, which suggested the protective role of miR‐128‐3p in HG‐induced DR inflammatory injury. Consistently, miR‐128‐3p overexpression has been reported to reverse HG‐induced angiogenesis in retinal vascular endothelial cells and alleviate HG‐triggered impairment of the blood‐retinal barrier [10]. Treatment with an miR‐128‐3p inhibitor was found to aggravate HG‐induced retinal pigment epithelial cell injury via activation of the Wnt/β‐catenin pathway [11]. Silencing of miR‐128‐3p was also reported to reverse the alleviation of hemin‐induced BV2 cell inflammatory response/neurotoxicity by PVT1 knockdown [14]. We report for the first time that miR‐128‐3p is involved in DR‐induced endothelial cell inflammatory injury by targeting KDM3A expression. All these findings suggest that targeting miR‐128‐3p may be a potential therapeutic modality for DR‐associated inflammatory injury in clinical practice.

With the aim of investigating the downstream mechanism of miR‐128‐3p, we obtained and confirmed the binding of miR‐128‐3p to the KDM3A 3′UTR through Starbase database prediction and dual‐luciferase assay. Histone demethylase KDM3A, a critical regulator of oxidative stress and inflammation‐related events, reacts to vascular injury in diabetes, leading to diabetes progression and vascular disorders [16, 29]. KDM3A drives oxidative stress, inflammatory response, apoptosis, and subsequent myocardial injury by mediating the transcription of NF‐κB/p65 in the diabetic heart [17]. Knockdown of KDM3A reduces oxidative stress and inflammatory injury in human umbilical vein endothelial cells under conditions of hyperglycemia and hypoxia [29]. In this study, a high expression profile of KDM3A was revealed in the HG group, and cells overexpressing with KDM3A and miR‐128‐3p showed significantly reduced viability and IL‐10 expression, but notably elevated TNF‐α and IL‐1β, suggesting that overexpression of KDM3A partially reversed the inhibitory effect of miR‐128‐3p overexpression on DR‐associated inflammatory injury.

KDM3A promotes the transcription of downstream target genes by the removal of H3K9me2 [26]. NLRP3 inflammasome is a multimeric cytosolic protein complex that plays a vital role in innate immunity. Activation of the NLRP3 inflammasome is crucial for pro‐inflammatory events in DR [30]. We speculated that KDM3A was involved in the DR inflammatory process by regulating NLRP3 expression. Results revealed that enrichment of KDM3A on the NLRP3 promoter was enhanced in HG‐treated cells but reduced after overexpression of miR‐128‐3p, while the enrichment of H3K9me2 on the NLRP3 promoter showed the opposite trend. In addition, transfection of oe‐KDM3A into cells overexpressing miR‐128‐3p markedly reduced the enrichment of H3K9me2 on the NLRP3 promoter while increasing the enrichment of KDM3A. The mRNA and protein levels of NLRP3 in the HG group were significantly higher than those in the control group. Overexpression of miR‐128‐3p reduced NLRP3 mRNA and protein levels, while these levels were increased following overexpression of KDM3A. These results indicated that KDM3A promoted the transcription and expression of NLRP3 via removal of histone H3K9me2. Previous studies have reported activation of NLRP3 following hyperglycemic stress in retinal cells [31]. NLRP3 activation not only stimulates the release of the inflammatory cytokines IL‐18 and IL‐1β but also induces pyroptosis, resulting in inflammation‐associated cell death of retinal cells and acceleration of DR progression [19]. Similarly, functional rescue experiments demonstrated that overexpression of NLRP3 partially reversed the inhibitory effect of miR‐128‐3p overexpression on DR inflammatory injury. Although the roles of KDM3A and NLRP3 in diabetes‐associated inflammation and oxidative stress have been reported, we propose for the first time that miR‐128‐3p targets KDM3A and then regulates NLRP3 expression through H3K9me2.

However, this study has several limitations. Specifically, only one target gene of miR‐128‐3p, KDM3A, was investigated, and it is possible that other miR‐128‐3p targets may be involved in the modulation of DR inflammatory injury. In addition, this study did not conduct in vivo experiments for verification. In the future, we will conduct in‐depth exploration of the role of other downstream target genes in DR and also include in vivo or patient‐sample validation in future work.

## 5 Conclusion

miR‐128‐3p reduces NLRP3 expression and thereby alleviates HG‐induced inflammatory injury in retinal endothelial cells via KDM3A‐mediated H3K9me2. This study is the first to propose the role of the KDM3A/H3K9me2/NLRP3 axis in DR inflammatory injury. These findings not only supplement the results of earlier research but also propose new ideas for the treatment of DR.

## Conflicts of Interest

The authors declare no conflicts of interest.

## Data Availability

The data that support the findings of this study are available from the corresponding author upon reasonable request.
